# Automated three-dimensional major white matter bundle segmentation using diffusion magnetic resonance imaging

**DOI:** 10.1007/s12565-023-00715-9

**Published:** 2023-04-05

**Authors:** Christina Andica, Koji Kamagata, Shigeki Aoki

**Affiliations:** 1 Faculty of Health Data Science, Juntendo University, 6-8-1 Hinode, Urayasu, Chiba 279-0013 Japan; 2 Department of Radiology, Juntendo University Graduate School of Medicine, 2-1-1 Hongo, Bunkyo-ku Tokyo, 113-8421 Japan

**Keywords:** Automatic, Diffusion magnetic resonance imaging, Tractography, White matter

## Abstract

White matter bundle segmentation using diffusion magnetic resonance imaging fiber tractography enables detailed evaluation of individual white matter tracts three-dimensionally, and plays a crucial role in studying human brain anatomy, function, development, and diseases. Manual extraction of streamlines utilizing a combination of the inclusion and exclusion of regions of interest can be considered the current gold standard for extracting white matter bundles from whole-brain tractograms. However, this is a time-consuming and operator-dependent process with limited reproducibility. Several automated approaches using different strategies to reconstruct the white matter tracts have been proposed to address the issues of time, labor, and reproducibility. In this review, we discuss few of the most well-validated approaches that automate white matter bundle segmentation with an end-to-end pipeline, including TRActs Constrained by UnderLying Anatomy (TRACULA), Automated Fiber Quantification, and TractSeg.

## Introduction

Diffusion magnetic resonance imaging (MRI) is an imaging method that measures the random microscopic motion (or diffusion) of water molecules in tissues, such as the white matter in the brain (Basser 1995). The highly coherent arrangement of white matter fiber tracts introduces the directional dependence of diffusion. For instance, the diffusion-driven displacement of water molecules in the white matter is less hindered along the orientation of the fibers than in their perpendicular direction (Moseley et al. 1990). This leads to anisotropic diffusion patterns that can be used to estimate the possible trajectories of white matter tracts noninvasively, which is known as fiber tracking or tractography (Mukherjee et al. 2008). In brief, tractography algorithms use estimates of the principal diffusion direction to trace the continuous trajectory of white matter fascicles (Basser et al. 2000; Mori et al. 1999).

White matter bundle segmentation, which is called “virtual dissection” using diffusion MRI fiber tractography, enables detailed evaluation of individual white matter tracts three-dimensionally (Catani et al. 2002). This approach plays a crucial role in the study of human brain anatomy and function. It also has enormous potential for the quantitative assessment (i.e., fiber count, fiber bundle volume, connectivity, and diffusion MRI measures) of white matter pathways in developing brains (Sonoda et al. 2021) or in cases involving neurological disorders (Ciccarelli et al. 2008; Kamagata et al. 2012, 2013a, b, 2014, 2018, 2019, 2020, 2021; Andica et al. 2018, 2020; Andica et al. 2021a, b). From a neurosurgical perspective, this approach has become a valuable tool for visualizing and localizing white matter tracts preoperatively and intraoperatively (Essayed et al. 2017).

Manual extraction of streamlines utilizing a combination of the inclusion and exclusion of regions of interest (ROIs) can be considered the current gold standard for extracting white matter bundles from whole-brain tractograms. However, this is a time-consuming and operator-dependent process with limited reproducibility (Rheault et al. 2020). Several automated approaches using different strategies to reconstruct the white matter tracts have been proposed to address the issues of time, labor, and reproducibility. Automated methods incorporating anatomical and prior orientational knowledge have considerably improved the accuracy of white matter tract segmentation (Rheault et al. 2019). Notably, these approaches are quick and address the issues of poor reproducibility and operator dependency in manual segmentation procedures (Kreilkamp et al. 2019).

In this review, we discuss few of the most well-validated approaches that automate white matter bundle segmentation from start to finish, including TRActs Constrained by UnderLying Anatomy (TRACULA) (Yendiki et al. 2011), Automated Fiber Quantification (AFQ) (Yeatman et al. 2012), and TractSeg (Wasserthal et al. 2018b, 2019). TRACULA, AFQ, and TractSeg are freely available as open-source pipelines for accurate white matter bundle segmentation from diffusion MRI. TRACULA uses prior knowledge of subcortical and cortical structures to automatically reconstruct 42 white matter bundles (Yendiki et al. 2011). AFQ uses the waypoint ROI procedure to identify 25 white matter bundles (Yeatman et al. 2012). Finally, TractSeg is a novel convolutional neural network-based approach that directly segments 72 white matter tracts in the fields of fiber orientation distribution function (FOD) peaks (Wasserthal et al. 2018b, 2019). These methods have been widely used in various brain disorders with promising results.

## TRACULA

TRACULA (Yendiki et al. 2011) automatically reconstructs major white matter bundles utilizing a global probabilistic approach based on the Bayesian framework (Jbabdi et al. 2007) to determine connections between predefined ROIs that best fit the preprocessed diffusion data. TRACULA adopts the ball-and-stick model (Behrens et al. 2007), which sets multiple anisotropic compartments per voxel, that is less sensitive to areas of high local uncertainty. Thus, TRACULA enables the estimation of areas with low anisotropy and tract crossings.

**Fig. 1:**
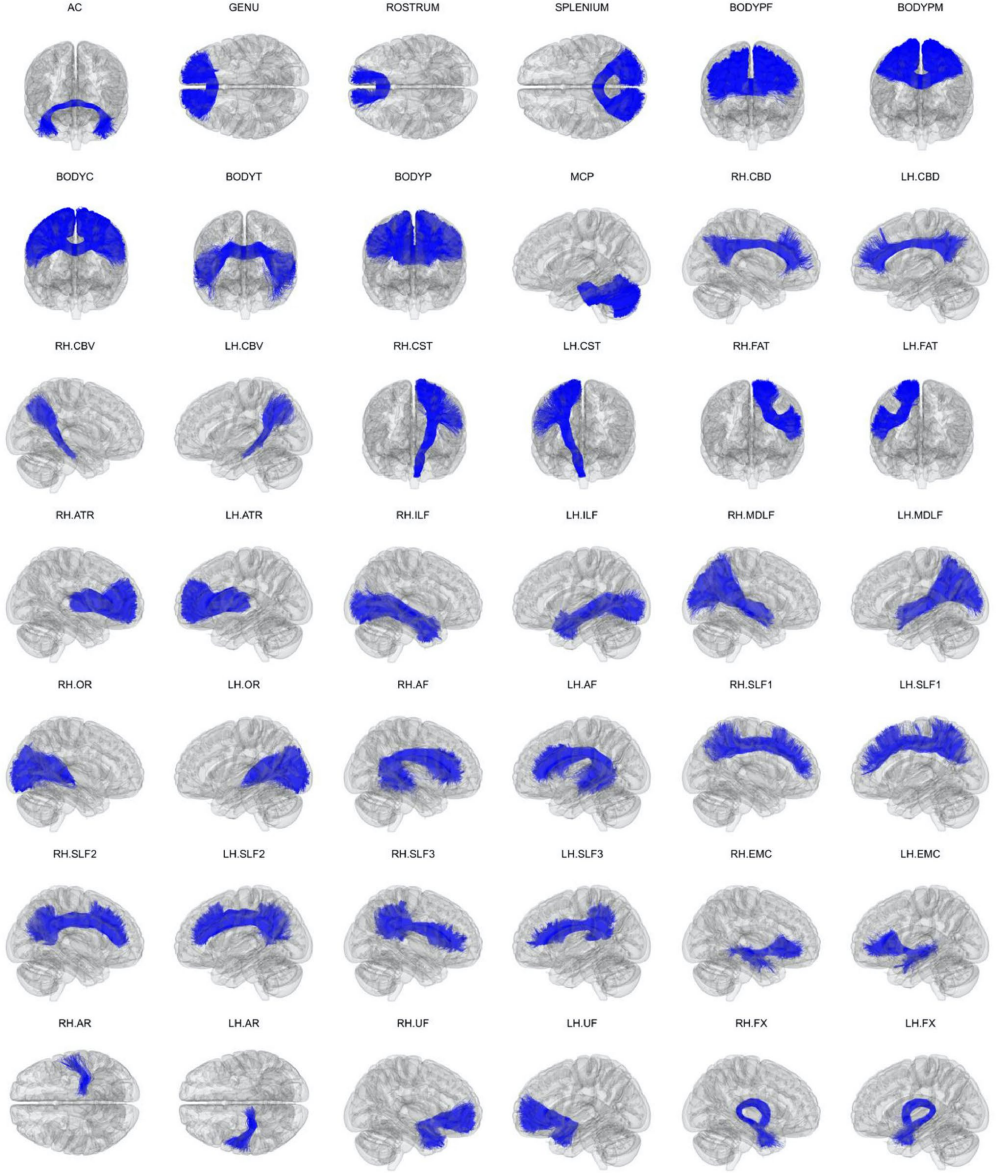
Manually labeled dataset for Tracts Constrained by Underlying Anatomy pipeline. Manually labeled streamlines from each of the 42 white matter bundles are shown aggregated over all 16 training subjects. Manual annotation was performed on each subject’s individual diffusion magnetic resonance imaging data as described previously (Maffei et al. 2021). Streamlines are displayed here in 1-mm MNI-152 template space. Adapted and reproduced with permission from (Maffei et al. 2021)

TRACULA is part of FreeSurfer (Fischl 2012), a widely used and freely available tool for analyzing neuroimaging data. TRACULA (Yendiki et al. 2011) incorporates prior information on possible white matter trajectories obtained from training subjects, where white matter tracts were labeled manually. TRACULA was initially trained on manual annotations of 18 white matter pathways (Yendiki et al. 2011), but now, TRACULA enables the reconstruction of 42 white matter pathways (Fig. 1) (Maffei et al. 2021) included in FreeSurfer 7.2 (https://github.com/freesurfer/freesurfer/tree/fs-7.2). Visualizations of the 42 manually annotated white matter bundles, as well as the along-tract profiles of microstructural measures on these bundles, are available at https://dmri.mgh.harvard.edu/tract-atlas/.

TRACULA uses the endpoints established in the training set’s tracts, expands the endpoints, and transforms them into each subject’s native space. Then, TRACULA establishes probabilistic streamlines accounting for the anatomical FreeSurfer (Fischl 2012) cortical parcellation and subcortical segmentation, and applies control points to dictate the curvature of the tract. This method does not presume exact tract spatial location or shape; thus, the trajectory of the tract is only restricted with respect to the surrounding anatomical structures. This allows for individual variations across subjects while still establishing the same tracts for comparison. Extensive documents, including tutorials, are available on the FreeSurfer wiki (https://surfer.nmr.mgh.harvard.edu/fswiki/Tracula).

TRACULA provides a dedicated pipeline for processing longitudinal diffusion MRI data. It reconstructs the white matter pathways of interest jointly from a subject’s data at all time points, rather than processing each time point independently (https://ftp.nmr.mgh.harvard.edu/pub/docs/TraculaNov2013/tracula.workshop.v.pdf).

The ongoing myelination of white matter fiber bundles plays a significant role in brain development. The reliable and consistent identification of these bundles on infant brain MRI is often challenging owing to inherently low diffusion anisotropy, as well as motion and other artifacts. Recently, TRActs Constrained by UnderLying Infant Anatomy (TRACULInA) was proposed for automatic white matter bundle segmentation in newborn brains (Zollei et al. 2019). In line with TRACULA, TRACULInA relies on global probabilistic tractography with prior knowledge on the anatomy of 14 manually annotated white matter pathways obtained from full-term and preterm infants (Zollei et al. 2019). However, to our knowledge, the TRACULInA algorithm is not yet freely available.

### TRACULA pipeline

**Fig. 2:**
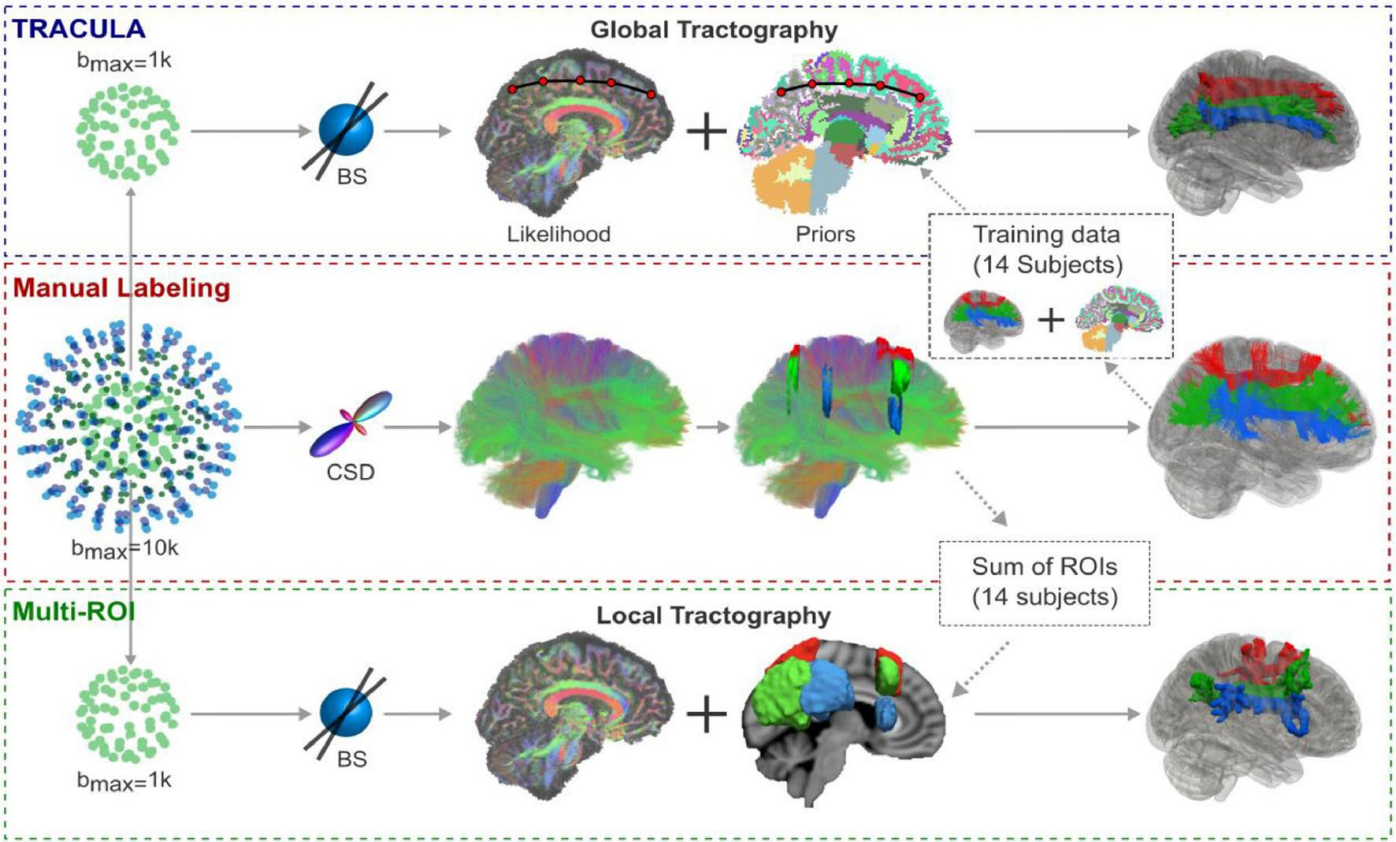
Overview of the Tracts Constrained by Underlying Anatomy (TRACULA) pipeline. From the four-shell MGH-USC Human Connectome Project data, the *b* = 10,000 s/mm^2^ and *b* = 1,000 s/mm^2^ shells were extracted. Orientations were reconstructed with constrained spherical deconvolution (CSD) from the *b* = 10,000 s/mm^2^ shell and with multi-shell multi-tissue CSD (MSMT-CSD) from all four shells. Streamline tractography was performed with these two approaches and used to annotate 42 tracts manually in 16 subjects. The lower shell (*b* = 1000 s/mm^2^, 64 directions) was used to reconstruct the same tracts automatically, with TRACULA or with a multi-region of interest (ROI) approach. For TRACULA, anatomical priors for each subject were obtained from the other 15 subjects and global probabilistic tractography was performed. For the multi-ROI approach, inclusion and exclusion masks were obtained from summing the manually defined ROIs of the other 15 subjects in template space. Local probabilistic tractography was constrained by these ROIs. The same ball-and-stick diffusion model was used for both TRACULA and the multi-ROI approach. Adapted and reproduced with permission from (Maffei et al. 2021)

The TRACULA “trac-all” (https://surfer.nmr.mgh.harvard.edu/fswiki/trac-all#Processingstepoptions) package automates the segmentation steps and reconstructs 42 major white matter pathways in the native diffusion space, with the following steps (Fig. 2):

1. Diffusion MRI preprocessing
  Preprocessing of the diffusion image data is needed before white matter pathways can be reconstructed by TRACULA, including compensation for B0 inhomogeneity and eddy current distortions, computing measures of head motion during the diffusion-weighted imaging (DWI) scan, intra-subject registration (individual DWI image to individual T1-weighted image), inter-subject registration (individual to a common template space), tensor fitting for extraction of diffusion tensor-based measures (i.e., fractional anisotropy [FA], mean diffusivity [MD], axial diffusivity [AD], and radial diffusivity [RD]), and computing anatomical priors for white matter pathways from a manually annotated set of training subjects.
2. BedpostX processing
  Bayesian estimation of diffusion parameters obtained using sampling techniques for modeling crossing fibers (BedpostX; https://fsl.fmrib.ox.ac.uk/fsl/fslwiki/FDT/UserGuide#BEDPOSTX), which is part of the FMRIB Software Library (Jenkinson et al. 2012), is used to fit the ball-and-stick model to the DWI data, estimating probability distributions of the diffusion signals at every voxel.
3. White matter pathway reconstruction
  The final step is to generate the probability distributions for all 42 white matter pathways in FreeSurfer 7.2 and 18 pathways in earlier FreeSurfer versions or a subset of pathways. This is performed by simultaneously fitting the shape of each pathway to the results of the ball-and-stick diffusion model from BedpostX and to the prior knowledge of the pathway anatomy obtained from the manually annotated set of training subjects comprising the TRACULA atlas.
4. Computing tract statistics
  Using TRACULA, it is possible to extract diffusion tensor-based measures (i.e., FA, MD, AD, and RD) either as average values over each pathway or as profiles along the trajectory of each pathway. Moreover, it is possible to adopt a general linear model for along-tract measures.

### TRACULA validation

TRACULA was initially trained to segment 18 white matter fiber bundles using low-quality and more widely available diffusion MRI data (i.e., 60 gradient directions, *b*-value = 700 s/mm^2^, and 10 *b* = 0 images) (Yendiki et al. 2011). Recently, TRACULA was trained on high-quality data (*b*_max_ = 10,000 s/mm^2^) from the Human Connectome Project (HCP; https://www.humanconnectome.org), and it showed an improvement in the accuracy of the reconstruction of 42 white matter tracts in routine-quality data (*b* = 1000 s/mm^2^) (Maffei et al. 2021).

TRACULA uses reproducible tracking protocols validated on a set of healthy training subjects, and it has been shown to be sensitive to white matter changes in patients, as long as the disease does not cause a radical reorganization of the brain and rerouting of white matter connections (Yendiki et al. 2011). The performance of TRACULA is independent of the method used for inter-subject registration (i.e., with or without affine inter-subject registration) (Maffei et al. 2021). This is because the anatomical priors in TRACULA only encode information about the relative positions (left, right, anterior, etc.) of the pathways with respect to their surrounding anatomical structures, not the absolute coordinates of the pathways in template space. Furthermore, the test–retest error in diffusion MRI measures measured across all 42 white matter bundles was lower with TRACULA (2.6–5.7%) than with a more conventional multi-ROI approach (12.4–17.0%) (Maffei et al. 2021).

White matter tracts may occasionally be partially reconstructed using TRACULA. A semiautomated quality control pipeline (He et al. 2021) for assessing and correcting possible errors that persist after running TRACULA should be considered to improve the detection and recovery of incomplete white matter tracts. The pipeline includes the following: (1) a visual inspection of eddy current-corrected diffusion-weighted images; (2) an automated evaluation of color-encoded FA images; (3) an assessment of the volume of each tract saved in the TRACULA output file; (4) reprocessing of tracts with a volume smaller than a specified threshold; (5) minimal manual editing of the control points for tracts that remained partially reconstructed; and (6) final reinitiation of TRACULA.

### TRACULA clinical applications

**Fig. 3:**
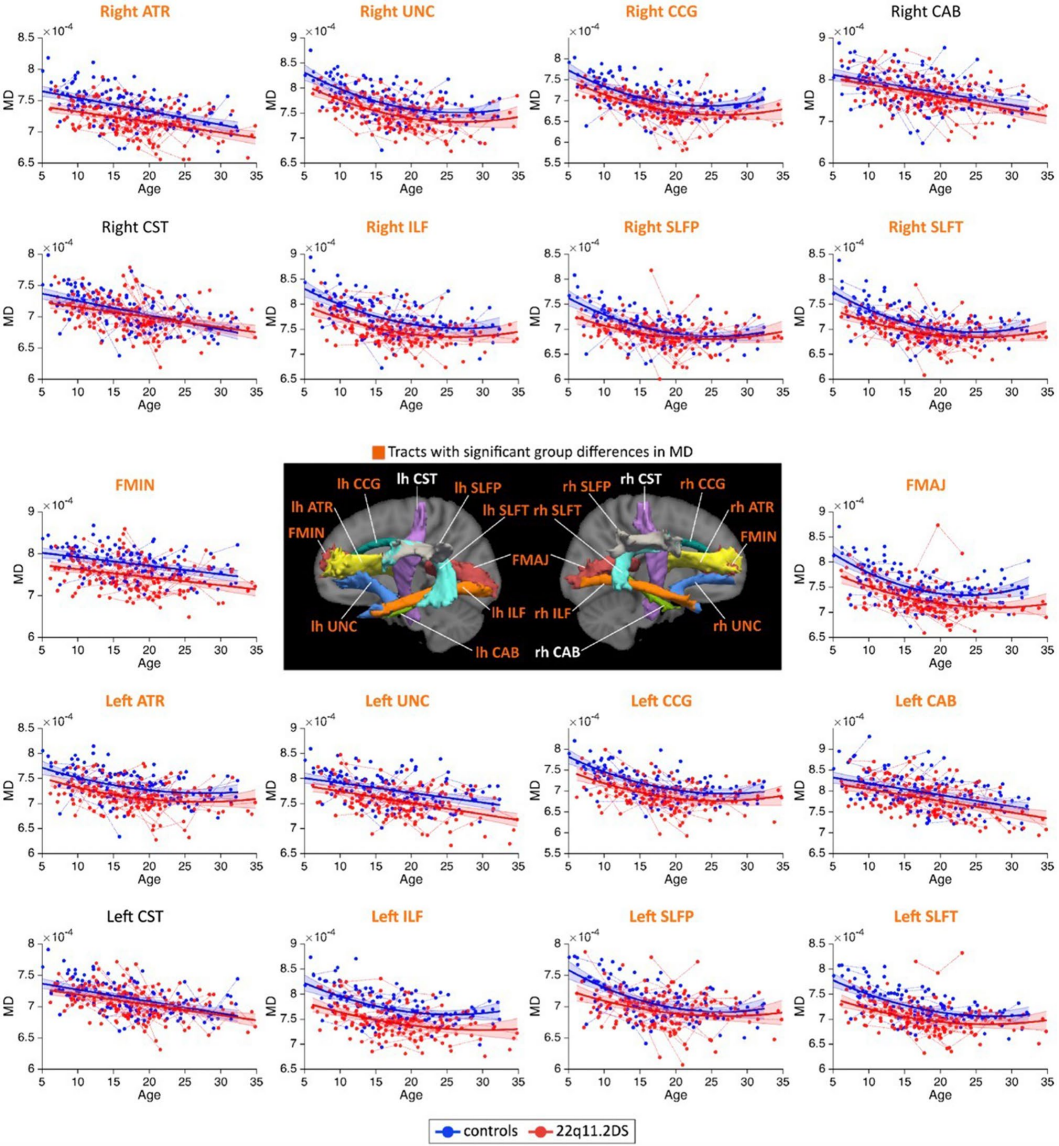
Developmental trajectories of mean diffusivity (MD) in patients with 22q11.2DS and controls assessed using the Tracts Constrained by Underlying Anatomy method. The MD metrics followed linear or quadratic decreasing trajectories in 22q11.2DS (red) and controls (blue). No significant age × group interaction effects were identified, indicating that patients with 22q11.2DS and controls followed parallel developmental curves with similar shapes. However, significant group differences were noted in MD, with systematic reductions in individuals with 22q11.2DS for most white matter tracts. Tracts sowing significant intergroup differences are highlighted in orange in plot titles and the central figure. A summary of all *p*-values for group and age × group interaction effects in white matter tracts has been presented in the Supplementary Materials, Table S4 in Bagautdinova (2020). The significance threshold was set at *p* < 0.05. All results were corrected for multiple comparisons using the false discovery rate method. *Lh* left hemisphere, *rh* right hemisphere, *FMAJ* forceps major (corpus callosum), *FMIN* forceps minor (corpus callosum), *ATR* anterior thalamic radiation, *CAB* cingulum–angular bundle, *CCG* cingulum–cingulate bundle, *CST* corticospinal tract, *ILF* inferior longitudinal fasciculus, *SLFP* superior longitudinal fasciculus–parietal bundle, *SLFT* superior longitudinal fasciculus–temporal bundle, *UNC* uncinate fasciculus. Adapted and reproduced with permission from Bagautdinova (2020)

**Fig. 4:**
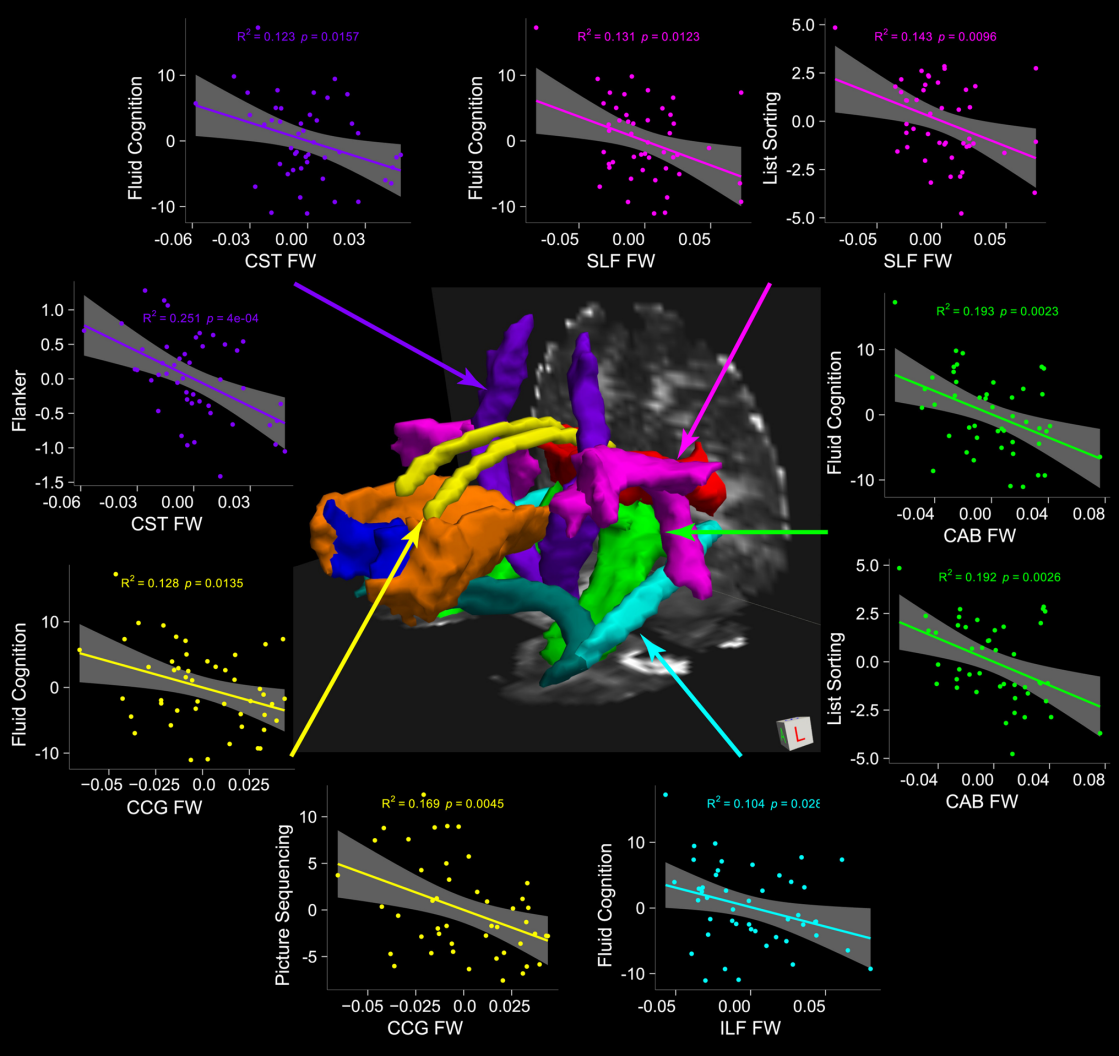
Tracts Constrained by Underlying Anatomy was utilized to produce major white matter pathways on which the diffusion metrics of white matter microstructure were overlaid and examined for their contribution to cognitive function independent of age, education, and sex. Note: Only significant associations are displayed. The current study used the 99% probability core white matter pathway for each tract of interest to minimize potential influence from cerebrospinal fluid. *FW* free water, *FA* fractional anisotropy, *FW-adjusted FA* free water-adjusted fractional anisotropy, *ATR* anterior thalamic radiation, *CAB* cingulum–angular bundle, *CCG* cingulum–cingulate bundle, *CST *corticospinal tract, *Fmajor* forceps major (corpus callosum), *Fminor* forceps minor (corpus callosum), *ILF* inferior longitudinal fasciculus, *SLF* superior longitudinal fasciculus, *UNC* uncinate fasciculus. Adapted and reproduced with permission from (Gullett et al. 2020)

TRACULA has been applied for various diseases, such as neuropsychiatric diseases (Bagautdinova et al. 2020 (Fig. 3); Ji et al. 2017; Mamah et al. 2019; Watanabe et al. 2018), epilepsy (Gharaylou et al. 2019; Kreilkamp et al. 2017), multiple sclerosis (Gharaylou et al. 2021), amyotrophic lateral sclerosis (Sarica et al. 2014), Parkinson’s disease (Pietracupa et al. 2018), and traumatic brain injury (Ueda et al. 2021), to demonstrate the changes in diffusion MRI measures when compared with healthy controls and to show the association between the measures and clinical scores. Besides the conventional diffusion tensor-based measures, the fractional volume of extracellular free water, which is obtained using a free water-corrected diffusion tensor imaging (DTI) technique (Pasternak et al. 2009), measured in white matter tracts segmented using TRACULA showed a significant correlation with cognitive function in older adults (Fig. 4) (Gullett et al. 2020). Longitudinal TRACULA has demonstrated age-related changes in diffusion MRI measures in specific white matter tracts (Storsve et al. 2016).

### AFQ

**Fig. 5:**
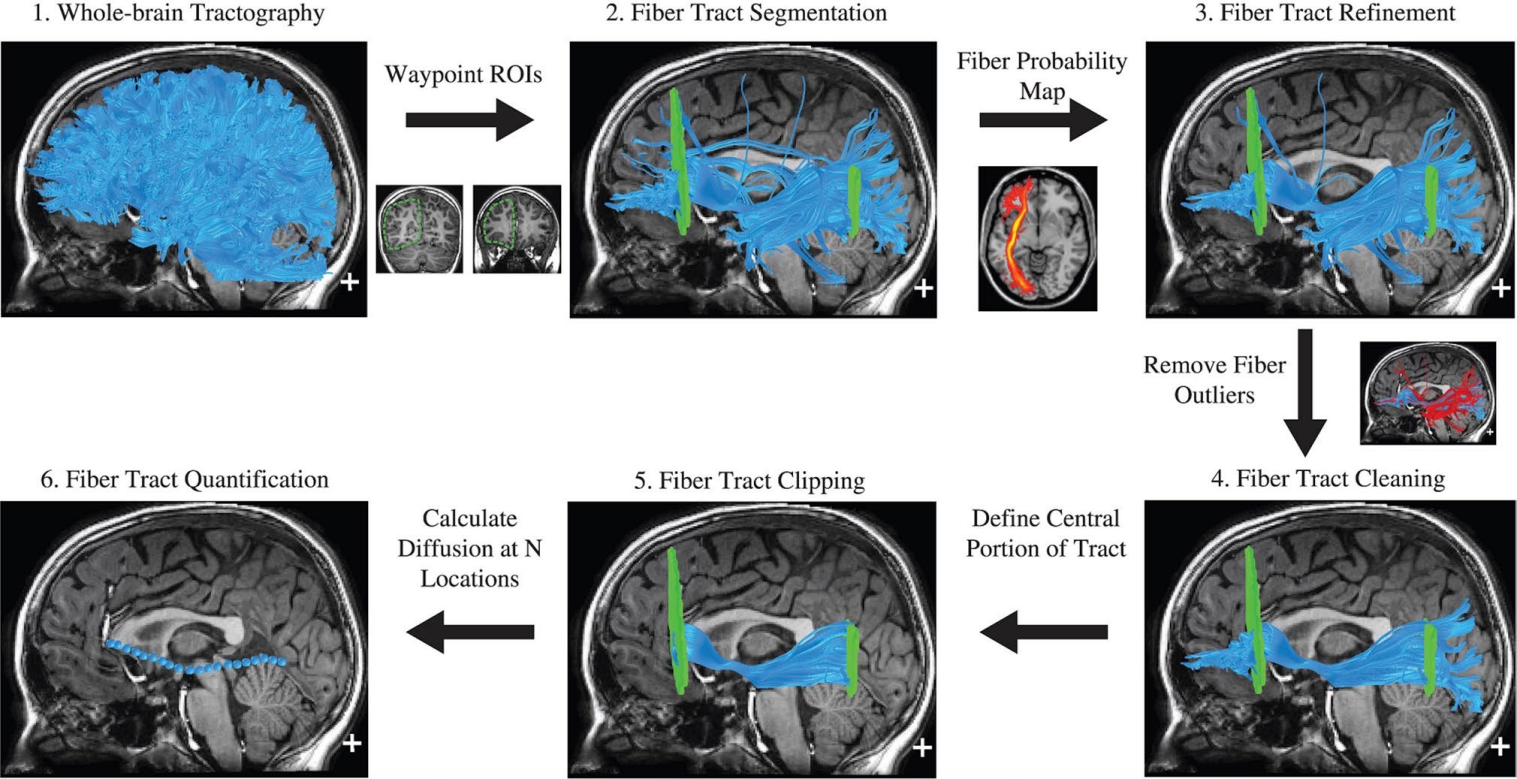
Automated Fiber Quantification procedure for the left hemisphere inferior fronto-occipital fasciculus. (1) Whole brain tractography is initiated from each white matter voxel with fractional anisotropy (FA) > 0.3. (2) Fibers that pass through two-waypoint regions of interest (ROIs) become candidates for the left inferior fronto-occipital fasciculus fiber group. (3) Each candidate fiber is then scored based on its similarity to a standard fiber tract probability map. Fibers with high probability scores are retained. (4) Fibers tracts are represented as a three-dimensional Gaussian distribution, and outlier fibers that deviate substantially from the mean position of the tract are removed. (5) The fiber group is clipped to the central portion that spans between the two defining ROIs. (6) The fiber group core is calculated by resampling each fiber into 100 equidistant nodes and calculating the mean location of each node. Diffusion measurements are calculated at each node by taking a weighted average of the FA measurements of each individual fiber’s diffusion properties at that node. Weights are determined based on the Mahalanobis distance of each fiber node from the fiber core. Adapted and reproduced with permission from (Yeatman et al. 2012)

AFQ is an open source software for automatically identifying white matter tracts and quantifying diffusion MRI measures at multiple locations along their trajectories (tract profiles) (Yeatman et al. 2012). In brief, AFQ uses a four-step procedure to identify white matter pathways in an individual’s brain, including whole-brain tractography, fiber tract segmentation with a two-waypoint ROI procedure, fiber tract refinement, and fiber tract cleaning (Fig. 5). Further details are provided in the “AFQ pipeline” section below.

**Fig. 6:**
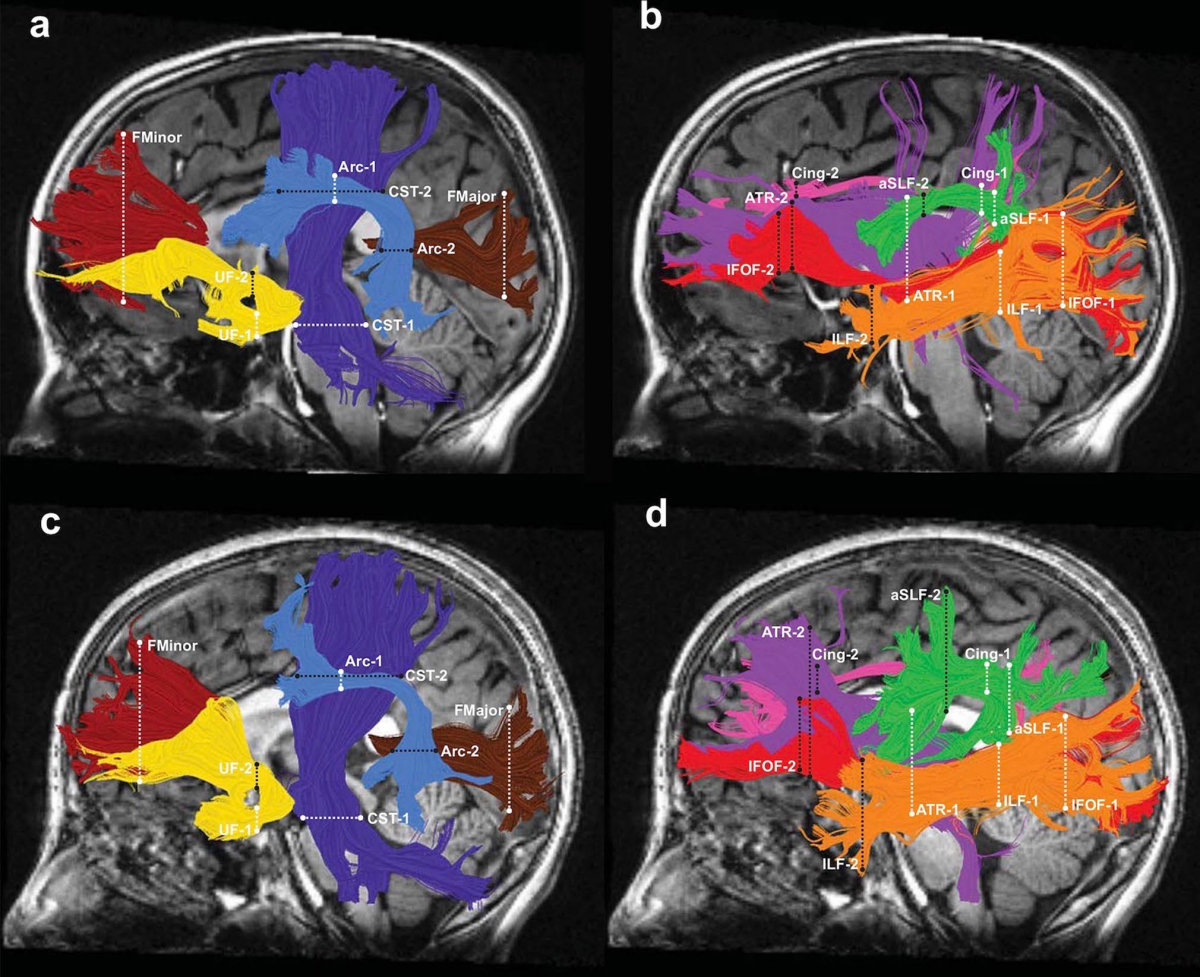
Tractography of 18 major cerebral white matter tracts constructed using Automated Fiber Quantification in preterm children and adolescents. The left hemispheric cerebral tracts are displayed on mid-sagittal T1 images from a representative full-term subject (**a**, **b**) and a representative preterm subject (**c**, **d**). Right hemisphere tract renderings are not shown. Panels a and c illustrate the following tracts: *Arc* arcuate fasciculus = light blue, *CST* corticospinal tract = dark blue, *Fmajor* forceps major = brown, *Fminor* forceps minor = dark red, and *UF* uncinate fasciculus = yellow. Panels b and d illustrate the following tracts: *ATR* anterior thalamic radiation = purple, *Cing* cingulum = magenta, *IFOF* inferior fronto-occipital fasciculus = red, *ILF* inferior longitudinal fasciculus = orange, and *aSLF* anterior superior longitudinal fasciculus = green. Dashed lines represent the location of the regions of interest (ROIs) used to isolate each cerebral tract (ROI 1 = white; ROI 2 = black). Adapted and reproduced with permission from (Travis et al. 2015)

AFQ has been released as open-source MATLAB (Yeatman et al. 2012, 2014a, b) and Python (Kruper et al. 2021) codes (https://github.com/yeatmanlab/AFQ). AFQ provides automatic segmentation of 18 white matter tracts (Fig. 6). Using the latest version of AFQ, it is possible to achieve the segmentation of the corpus callosum (i.e., posterior parietal, motor, orbitofrontal, temporal, superior frontal, and superior parietal callosum) (Yeatman et al. 2014a), cerebellar peduncles (Borchers et al. 2022), and vertical occipital fasciculus “VOF toolbox,” which is the only major fiber bundle connecting the dorsolateral and ventrolateral visual cortex (Yeatman et al. 2014a, b).

### AFQ pipeline

The AFQ pipeline involves the following aspects:

1. Diffusion MRI preprocessing
  The AFQ pipeline does not perform preprocessing steps; however, preprocessing software tools, including VISTASOFT (https://github.com/vistalab/vistasoft), dMRIPrep (https://github.com/nipreps/dmriprep), and QSIprep (https://qsiprep.readthedocs.io/en/latest/), have been utilized to preprocess diffusion data used in the AFQ pipeline (https://yeatmanlab.github.io/pyAFQ/usage/data.html).
2. Fiber tractography
  By default, whole-brain fiber tracts are estimated using a deterministic streamline tracking algorithm (Basser et al. 2000; Mori et al. 1999) with a fourth-order Runge–Kutta path integration method and a 1-mm fixed-step size. The tracking algorithm is seeded with a white matter mask defined as all the voxels with an FA value greater than 0.3. Individual streamline is terminated if the FA value at the current position is below 0.2 and the minimum angle between the last path segment and the next step is greater than 30°. This step can be performed with different fiber orientation estimation methods (i.e., constrained spherical deconvolution [CSD] (Yeatman et al. 2014b)) and tractography algorithms (https://yeatmanlab.github.io/pyAFQ/usage/converter.html).
3. Fiber tract segmentation
  The segmentation is based on the waypoint ROI procedure (Wakana et al. 2007). In brief, white matter fibers are assigned to a particular fiber group if they pass through two-waypoint ROIs that define the trajectory of the fascicle. The ROIs are defined in locations that isolate the central portion of the tract where the fibers are coherently bundled together and before they begin diverging toward the cortex. The ROIs are transformed into an individual’s native space based on an estimated nonlinear transformation to the MNI template space.
4. Fiber tract refinement
  In this step, the segmented fiber tracts are compared to fiber tract probability maps. The fiber tract probability maps of major fascicles were created by manually segmenting and coregistering each fiber group for 28 healthy adult subjects and calculating the proportion of subjects with a given tract in each voxel (Hua et al. 2008). The fiber tract probability maps are transformed into an individual’s native space and the segmented fibers for a particular fiber group are scored based on the probability values of the voxels they pass through. Segmented fibers with a low probability score are then discarded.
5. Fiber tract cleaning
  White matter fibers that are more than 4 standard deviations above the mean fiber length or that deviate more than 5 standard deviations from the core of the fiber tract are removed. AFQ version 1.1. (Yeatman et al. 2014a) includes an additional tract cleaning procedure in which fibers with aberrant cortical endpoints are removed by warping the cortical labels from the automated anatomical labeling atlas (Tzourio-Mazoyer et al. 2002) to an individual’s native space. This feature ensures that each fiber in the group starts and ends within 4 mm of its known cortical destination.
6. Fiber tract quantification
  The locations of the waypoint ROIs are used to isolate the central trajectory of the white matter bundle (Fig. 7), and this is followed by resampling each fiber to 100 equally spaced nodes (tract profiles). Diffusion properties are calculated at each node of each fiber using spline interpolation of the diffusion properties (FA, MD, AD, and RD).

**Fig. 7:**
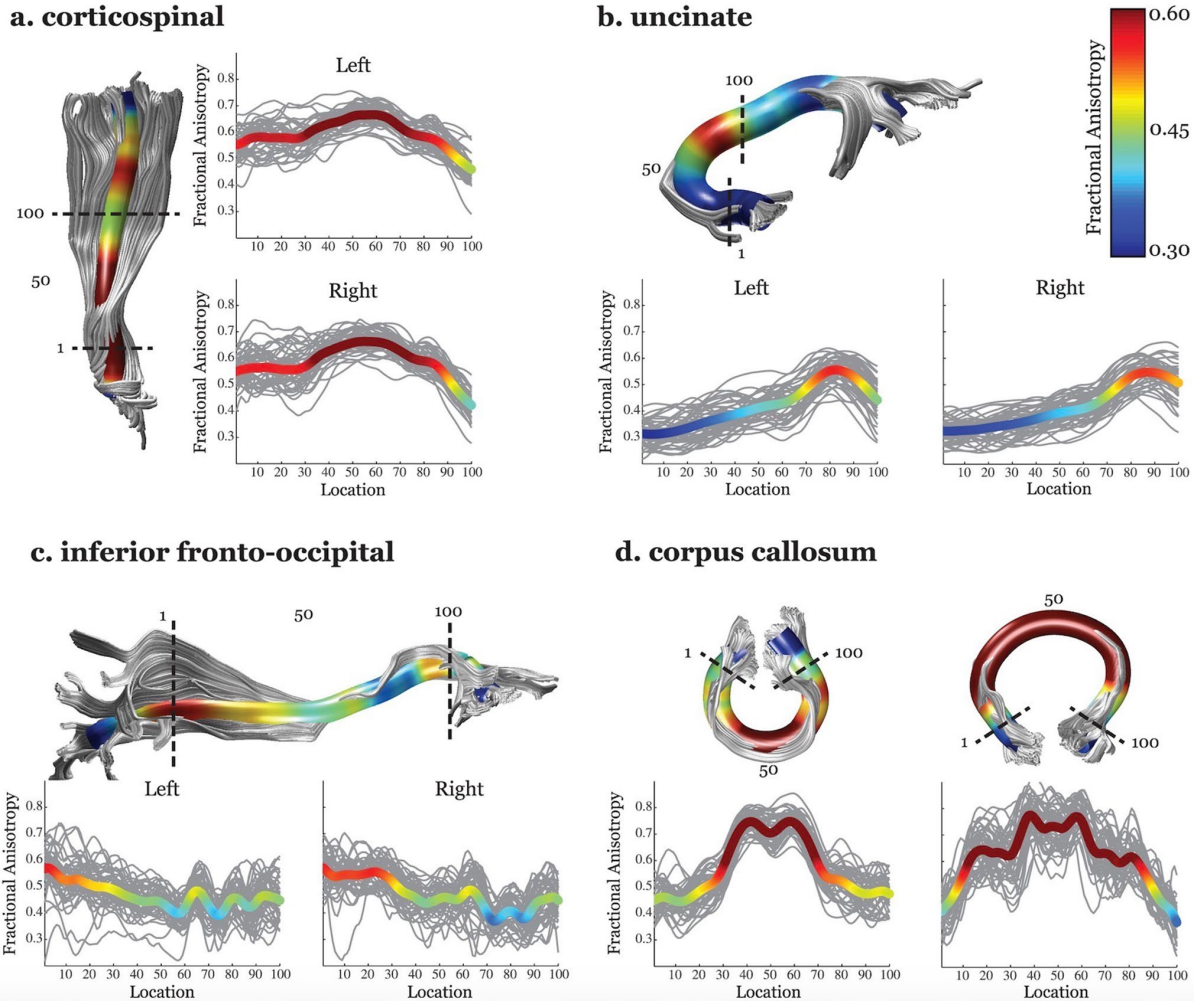
Tract fractional anisotropy (FA) profiles of four major fascicles (**a** corticospinal tract, **b** uncinate fasciculus, **c** inferior fronto-occipital fasciculus, and **d** corpus callosum) generated using Automated Fiber Quantification (AFQ). A tract profile quantifies diffusion measures at multiple locations along the trajectory of a white matter tract. For each tract, a three-dimensional rendering derived from the AFQ software is shown for a single representative 12-year-old female. It indicates the defining regions of interest (ROIs) as dotted lines and includes the core or mean fiber, represented as a 5-mm-radius tube color coded based on the FA value at each point along the tract for that subject. Adjacent to the rendering, tract FA profiles for the left and right hemispheres show the FA along the core fiber (*y*-axis) at each of 100 equidistant points (*x*-axis) along the fascicle between the defining ROIs for typically developing children and adolescents aged 9 to 16 years (*N* = 48). The group mean is shown as a bold line color coded based on the group mean FA value at that point. Tract FA profiles show a consistent pattern of peaks and valleys of FA across individuals. Adapted and reproduced with permission from (Yeatman et al. 2012)
7. Individual- and group-level inference
  Standardized tract profiles can be created using the mean and standard deviation of each diffusion property at each node of each tract. Statistical analysis can be performed point-wise along the tract profiles.

### AFQ validation

AFQ has been validated using clinical quality (i.e., 30 gradient directions, *b*-value = 900 s/mm^2^, and 10 *b* = 0 images) (Yeatman et al. 2012) and HCP quality (Kruper et al. 2021) diffusion MRI data. The test–retest reliability of tractometry performed using low- and high-quality diffusion MRI data was relatively high, but it was higher using high-quality data. The tractometry results were highly consistent between different FOD models used in tractography (i.e., DTI- and CSD-derived FODs) software implementations (i.e., MATLAB and Python AFQ) (Kruper et al. 2021). Furthermore, AFQ has been shown to have compatible correlation with the manual approach in the reconstruction of white matter tracts (Kreilkamp et al. 2019).

### AFQ clinical applications

**Fig. 8:**
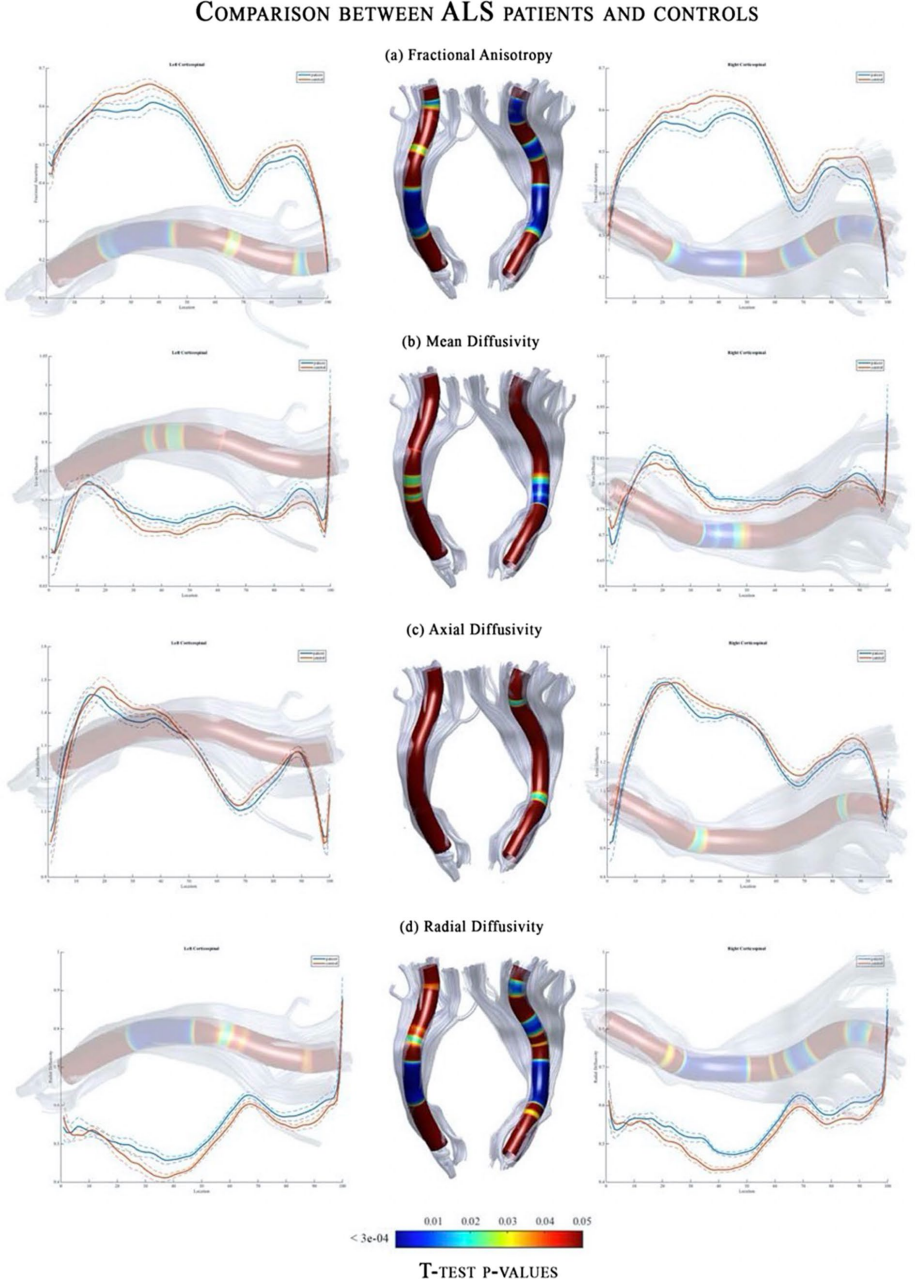
Diffusion tensor imaging-related profiles of the corticospinal tract (CST) in patients with amyotrophic lateral sclerosis compared controls obtained using automated fiber quantification (AFQ) pipeline. Plots of mean values are reported in a voxel-by-voxel manner for each group (patients in blue and controls in orange). Dotted lines ± 1 SD represent the left and right CST. The *x*-axis represents the voxel location (1–100) and the *y*-axis reports the subjects’ group mean values of **a** fractional anisotropy, **b** mean diffusivity, **c** axial diffusivity, and **d** radial diffusivity. *T*-test statistics are plotted using a 3D rendering derived from the AFQ software. A conventional criterion was used to threshold the results for multiple comparisons, which implied an appropriate threshold of significance at* P* < 3e − 04. The 3D representation (glass effect) of the tract is added to the plot background so that each location reported on the *x*-axis corresponds to the same location in the 3D view. In the middle part of the figure, the same 3D representation is reported for the left and right tract where the *P*-values are associated with colors of a heat map (statistically significant differences are shown in blue). Adapted and reproduced with permission from (Sarica et al. 2017)

AFQ has been applied to show the changes in diffusion tensor-based measures along white matter fiber tract in cerebral small vessel disease (Huang et al. 2020), epilepsy (Kreilkamp et al. 2019), Alzheimer’s disease (Zhang et al. 2019; Xue et al. 2019; Dou et al. 2020; Chen et al. 2020), neuropsychiatric disorders (Sacchet et al. 2014a, b; Zhang et al. 2022a), neuromyelitis optica spectrum disorder (Yan et al. 2022), fragile X syndrome (Hall et al. 2016), and amyotrophic lateral sclerosis (Sarica et al. 2017) (Fig. 8), as well as in obese adolescents (Carbine et al. 2020), children with attention-deficit/hyperactivity disorder (Lin et al. 2020) and dyslexia (Banfi et al. 2019), and neonates at risk of stuttering (Packman et al. 2022). Moreover, AFQ has shown that long-term cognitive activities, such as chess, may systematically influence the white matter properties of memory, attention, and visual pathways (Zhou et al. 2018). Furthermore, AFQ has demonstrated changes in diffusion kurtosis imaging (Jensen et al. 2005) measures in patients with epilepsy (Kasa et al. 2022) and age-related changes of R1 (1/T1), an indicator of myelin content (Yeatman et al. 2014a).

## TractSeg

TractSeg is a novel, fully automatic approach for direct white matter tract segmentation (Wasserthal et al. 2018b, 2019). This approach is based on a fully convolutional neural network architecture (U-Net) that directly segments white matter tracts in fields of FOD peaks. TractSeg does not require an intensive processing pipeline, such as affine or elastic registration, parcellation, or clustering, shortening the runtime (around 20.1 min).

**Fig. 9:**
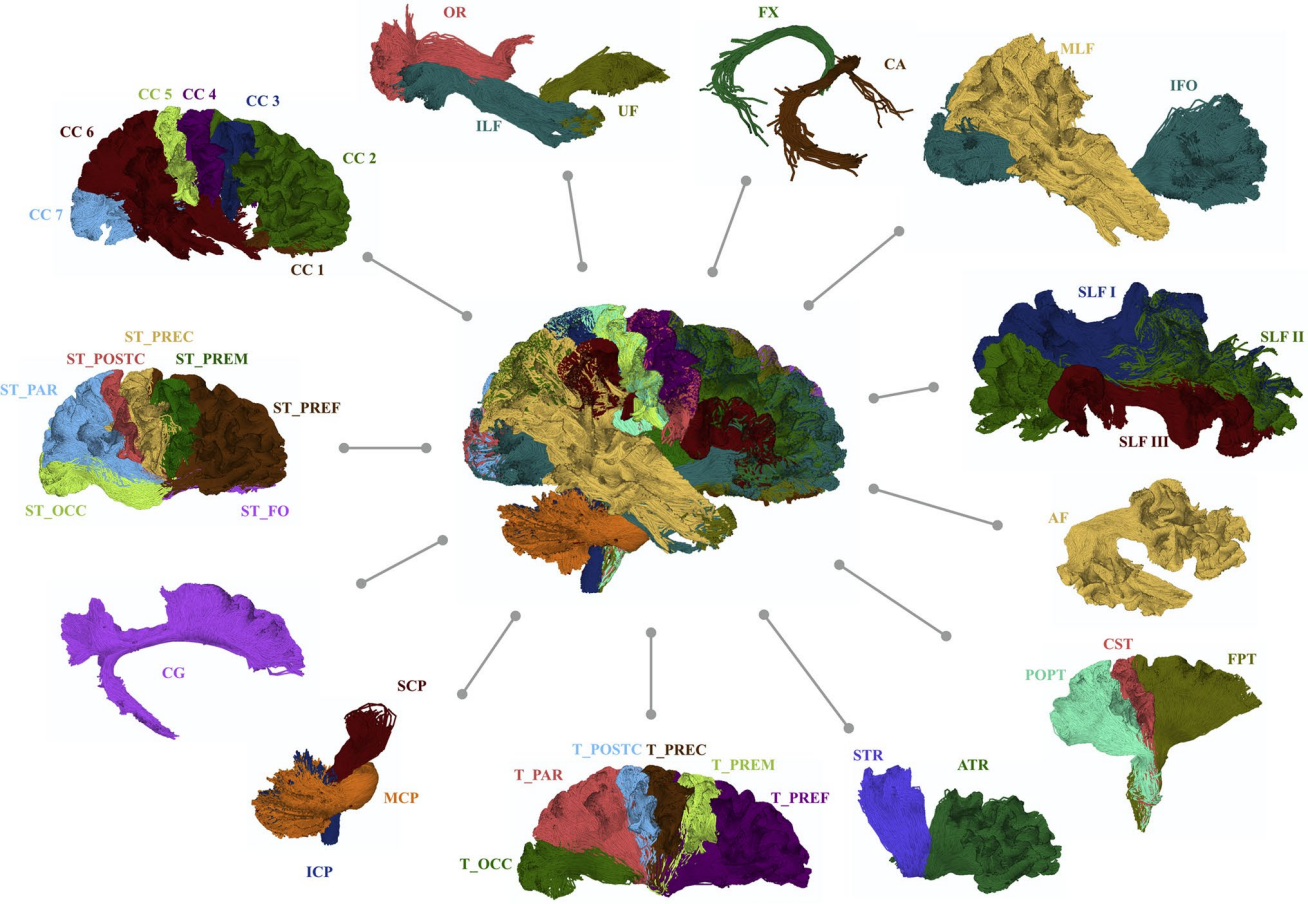
Overview of all 72 white matter tracts constructed using TractSeg. For tracts that exist in the left and right hemispheres, only the right one is shown. The following tracts are included: *AF* arcuate fascicle, *ATR* anterior thalamic radiation, *CA* commissure anterior, corpus callosum (CC1, rostrum, CC2, genu, CC3, rostral body, CC4, anterior midbody, CC5, posterior midbody, CC6, isthmus; and CC7, splenium), *CG* cingulum (CG), *CST* corticospinal tract, *MLF* middle longitudinal fascicle, *FPT* fronto-pontine tract, *FX* fornix, *ICP* inferior cerebellar peduncle, *IFO* inferior occipito-frontal fascicle, *ILF* inferior longitudinal fascicle, *MCP* middle cerebellar peduncle, *OR* optic radiation, *POPT* parieto-occipital pontine, *SCP* superior cerebellar peduncle, *SLF I* superior longitudinal fascicle I, *SLF II* superior longitudinal fascicle II, *SLF III* superior longitudinal fascicle III, *STR* superior thalamic radiation, *UF* uncinate fascicle, *T_PREF* thalamo-prefrontal, *T_PREM* thalamo-premotor, *T_PREC* thalamo-precentral, *T_POSTC* thalamo-postcentral, *T_PAR* thalamo-parietal, *T_OCC* thalamo-occipital, *ST_FO* striato-fronto-orbital, *ST_PREF* striato-prefrontal, *ST_PREM* striato-premotor, *ST_PREC* striato-precentral, *ST_POSTC* striato-postcentral, *ST_PAR* striato-parietal, and *ST_OCC* striato-occipital. Adapted and reproduced with permission from (Wasserthal et al. 2018b)

TractSeg used a semiautomatic approach to generate binary reference segmentations for 72 anatomically well-described white matter tracts (Fig. 9) for each subject in a cohort of 105 healthy young subjects selected from the HCP (Wasserthal et al. 2018b). These reference segmentations were used as labels for training and validating the algorithm. More details on the white matter bundle segmentation processing steps are available in a previous report (Wasserthal et al. 2018b).

### TractSeg pipeline

**Fig. 10:**
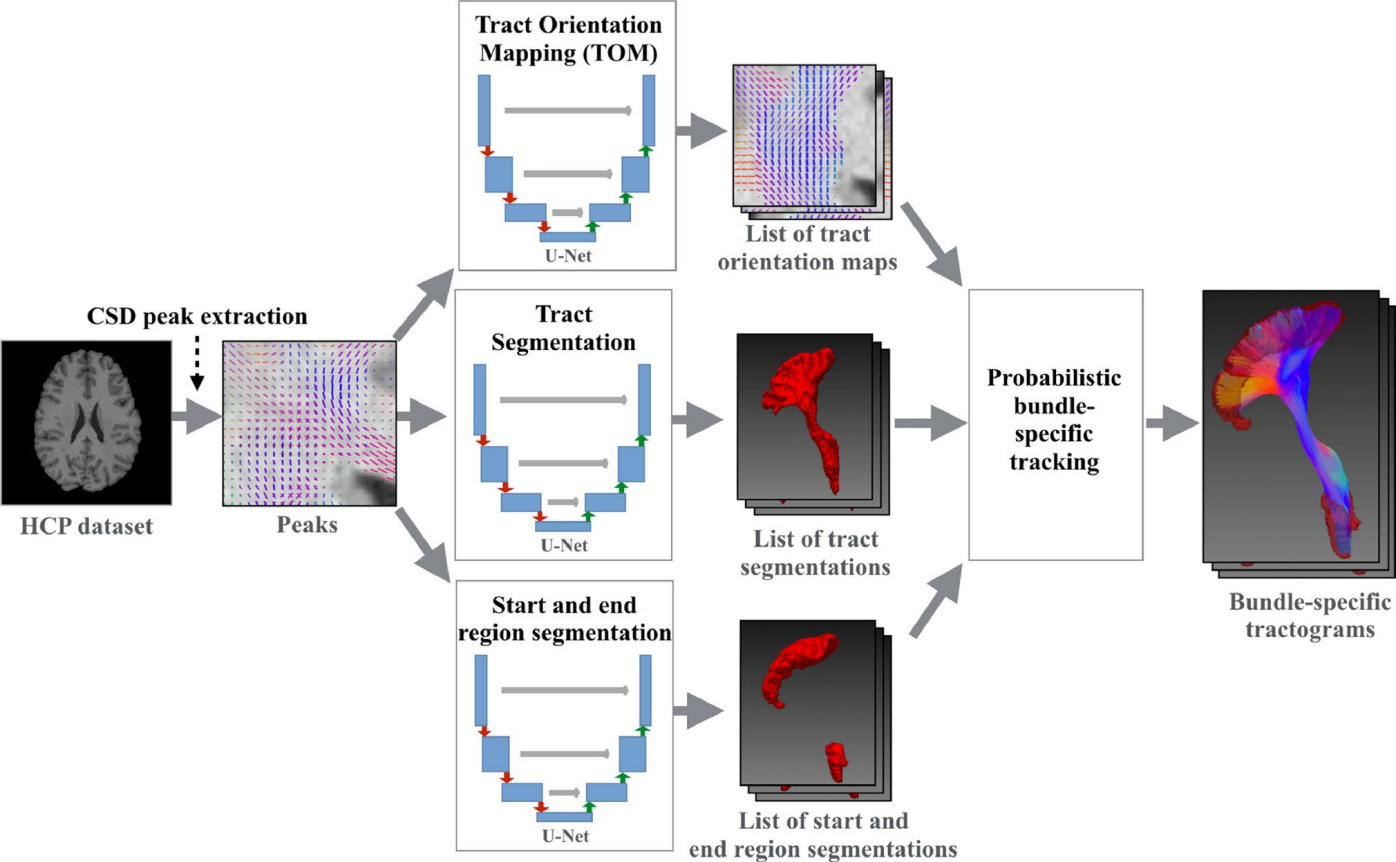
TractSeg pipeline overview. Constrained spherical deconvolution is applied to obtain the three principal fiber orientation distribution directions per voxel, which is the input for three U-Nets. The three U-Nets are used to create a tract orientation map, a tract mask, and a start/end region mask for each tract. Then, probabilistic tracking is run on the tract orientation maps. All streamlines leaving the tract mask and not ending in the start/end masks are discarded. The result is one tractogram for each tract. Adapted and reproduced with permission from (Wasserthal et al. 2019)

The TractSeg code is openly available as an easy-to-use Python package with pretrained weights (https://github.com/MIC-DKFZ/TractSeg/). The pipeline of TractSeg can be summarized as follows (Fig. 10):

1. Diffusion MRI preprocessing steps
  There is no definite diffusion MRI preprocessing pipeline required for TractSeg. However, TractSeg has been trained using HCP minimal preprocessed diffusion MRI data (i.e., distortion correction, motion correction, registration to MNI space, and brain extraction) (Wasserthal et al. 2018b).
2. Extraction of FOD peaks
  The principal fiber directions are estimated from the diffusion data using the CSD and peak extraction available in MRtrix (Jeurissen et al. 2014; Tournier et al. 2007, 2019). Three principal fiber directions per voxel are then obtained as input for U-Nets (Wasserthal et al. 2019). The TractSeg algorithm runs well with multi-shell multi-tissue or single-shell CSD peaks because both types of data were sampled to train the network (Wasserthal et al. 2019). TractSeg also works with BedpostX peaks as input. Further details can be obtained from https://github.com/MIC-DKFZ/TractSeg#how-to-use.
3. Tract orientation mapping
  The original FOD peaks are used to produce tract orientation maps (Wasserthal et al. 2018a). Each tract orientation map represents one tract, and each voxel contains one orientation vector representing the local tract orientation, that is, the local mean streamline orientation of the tract. Fiber tracking on the tract orientation maps has been shown to have high sensitivity (tract is complete) and specificity (few false positives) (Wasserthal et al. 2019).
4. Tract segmentation
  Segmentation masks of 72 white matter fiber bundles are generated for each subject (Wasserthal et al. 2018b).
5. Start and end region segmentation
  All streamlines not ending in the start/end regions are removed (Wasserthal et al. 2019).
6. Probabilistic bundle-specific tracking
  Probabilistic fiber tracking is run on the tract orientation maps. The probabilistic approach to tract orientation mapping tractography maximizes the sensitivity of the bundle-specific tractography, even on low-resolution data or strongly bent tracts (Wasserthal et al. 2019).
7. Tractometry
  After the generation of specific tractograms using TractSeg, it is possible to evaluate diffusion MRI measures, such as fractional anisotropy, along 100 points of each tract (tractometry). Statistical analyses, such as *t*-tests and correlation analyses, can be performed point-wise along the 100 points (Wasserthal et al. 2020).

### TractSeg validation

For evaluating the segmentation performance of TractSeg, the Dice score was calculated for each subject from the training cohort between each of the 72 reference tracts constructed using HCP quality data (1.25-mm isotropic resolution, 270 gradient directions, three *b*-values [1000, 2000, and 3000 s/mm^2^], and 18 *b* = 0 images), clinical quality HCP data (HCP down-sampled data: 2.5-mm isotropic resolution and 32 gradient directions at *b* = 1000 s/mm^2^), and “FiberFox” phantom data (2.5-mm isotropic resolution and 32 gradient directions at *b* = 1000 s/mm^2^, with several artifacts) (Wasserthal et al. 2019). It has been found that the Dice scores of HCP quality data (0.85), clinical quality HCP data (0.80), and phantom data (0.74) were significantly higher (*P* < 0.01) than the scores of other methods, such as RecoBundles (0.67) (Garyfallidis et al. 2018) and TractQuerier (0.59) (Wassermann et al. 2016). These results indicate that the TractSeg algorithm is quite robust to lower image quality. A recent study has also demonstrated that TractSeg was feasible with *b* = 800 s/mm^2^ and data of 15 gradient directions (Tallus et al. 2022).

Furthermore, the TractSeg pipeline was tested on 17 differently acquired datasets (various scanners, various spatial resolutions, various *b*-values, different gradients, healthy and diseased cases, and normal and abnormal brain anatomy cases) (Wasserthal et al. 2019). TractSeg demonstrated anatomically plausible results for all subjects and most of the tracts. Nevertheless, small bundles, such as the anterior commissure (CA), were not wholly reconstructed in around 20% of subjects (Wasserthal et al. 2019). These results indicate that extra care is needed for small or thin bundles, such as the CA and fornix. To improve the segmentation of small bundles TractSeg provides a “super resolution” option (https://github.com/MIC-DKFZ/TractSeg#small-bundles-like-the-ca-and-fx-are-incomplete). This option can be applied to up-sample the input image to a resolution of 1.25 mm (the resolution TractSeg was trained on) and finally down-sample it back to the original resolution.

### TractSeg clinical applications

**Fig. 11:**
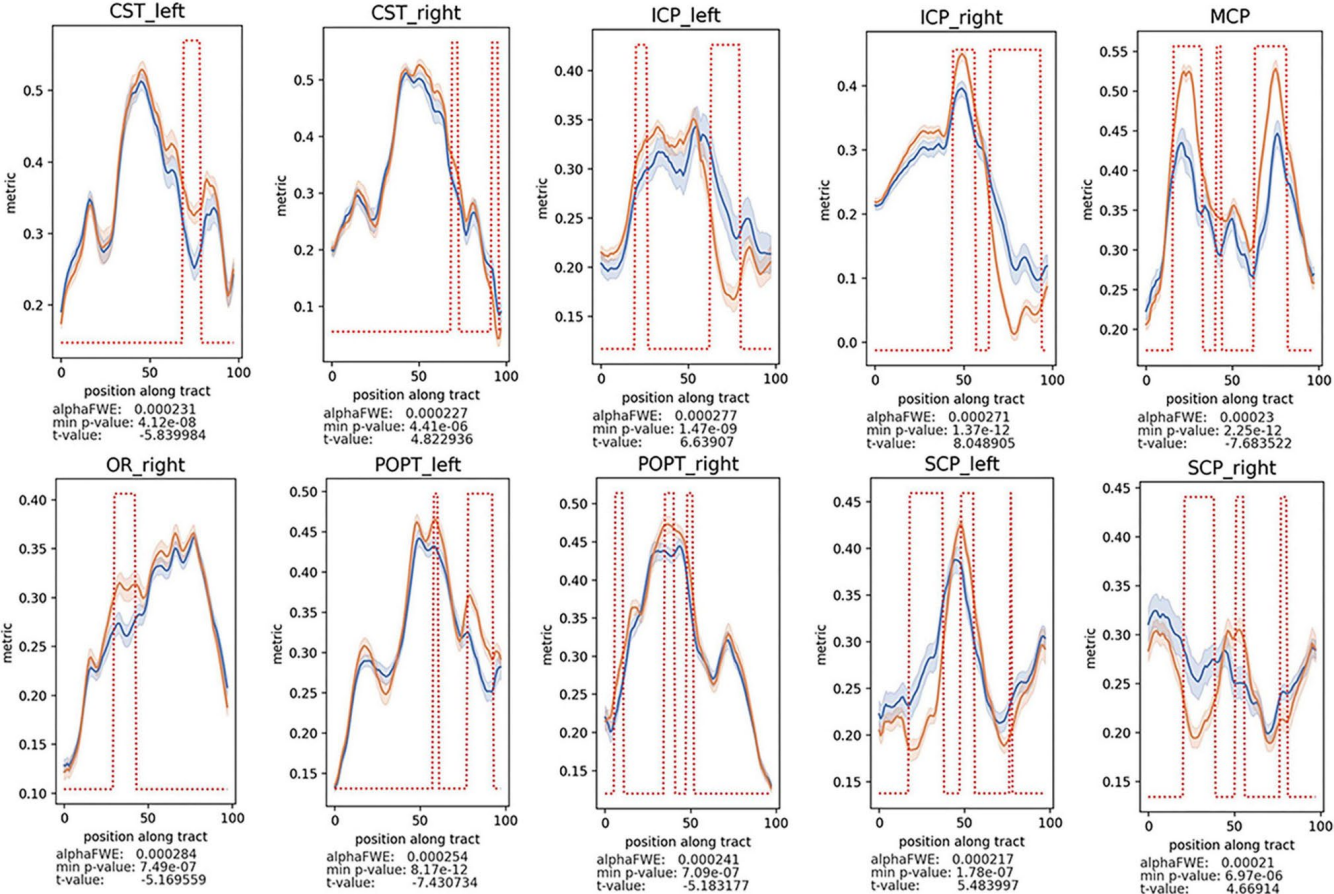
Fractional anisotropy (FA) differences between patients with brainstem glioma and controls evaluated using TractSeg. Decreased FA values of the *CST* cerebrospinal tract, *MCP* middle cerebellar peduncle, *ICP* inferior cerebellar peduncle, *OR* optic radiation, *POPT* parieto-occipital pontine tract, and *SCP* superior cerebellar peduncle, were found in patients vs. healthy controls (*P* < 0.05, family-wise error [FWE]-corrected). The alphaFWE is the alpha value corrected for multiple comparisons (multiple parts per tract and multiple tracts). The min *P*-value is the minimal *P*-value calculated for each tract. If min *P*-value < alphaFWE, the tract contains significant results. The red dotted lines above the lines of the patients’ group and the controls indicates all positions with the tract for which the *P*-value is < alphaFWE. Adapted and reproduced with permission from (Zhang et al. 2022b)

TractSeg has been applied to assess various neurological disorders, including amyotrophic lateral sclerosis (Tu et al. 2020), schizophrenia spectrum disorders (Wasserthal et al. 2020, 2021), traumatic brain injury (Tallus et al. 2022), ischemic stroke (Egorova-Brumley et al. 2022), and pediatric brainstem glioma (Zhang et al. 2022b) (Fig. 11), and it shows changes in diffusion MRI measures in individual white matter fiber bundles in patients compared with healthy controls.

**Fig. 12:**
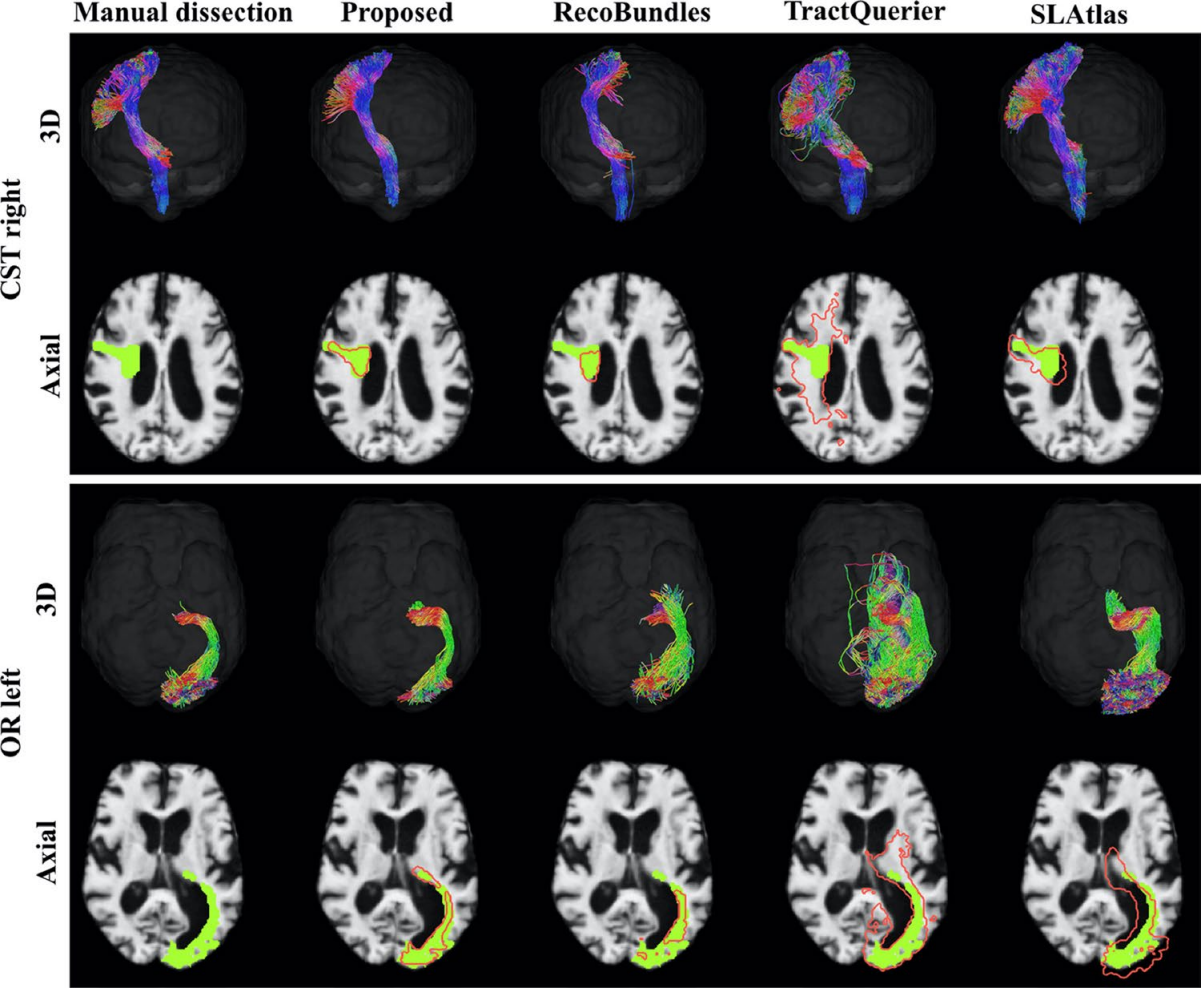
Qualitative comparison of results on one Alzheimer’s patient with enlarged ventricles from the OASIS dataset. Reconstruction of the right corticospinal tract (CST) and left optic radiation (OR). Green shows manual dissection, and red shows the tract mask of the respective method (proposed represents the results obtained using TractSeg). Please refer to the web version of this article for interpretation of the references to color in this figure legend. Adapted and reproduced with permission from (2018b (Wasserthal et al. 2019)

TractSeg-based corticospinal tract segmentation was implemented in 28 patients with brain masses adjacent to or displacing the corticospinal tract, and the automated algorithm was able to segment the bilateral corticospinal tracts (CSTs) in all patients, whereas the manual fiber segmentation approach failed to reconstruct the CSTs in 2 patients (Richards et al. 2021). In a large cohort (*N* = 625) of patients with various brain pathologies, TractSeg showed superior consistency in CST segmentation compared with the manual approach (Moshe et al. 2022). TractSeg was also able to perform fiber bundle segmentation in the presence of brain abnormalities, such as enlarged ventricles (Fig. 12), severe lesions in the pathways, or brain atrophy, similar to the manual approach and better compared with other methods (Wasserthal et al. 2019). Furthermore, TractSeg was able to segment the core of the acoustic radiation, a relatively short fiber bundle (4–6 cm), in patients with congenital ear canal atresia (Siegbahn et al. 2022).

DTI is currently the most widely used tool for assessing white matter microstructural alterations. However, it is limited by its inability to represent the diffusion signal in crossing or kissing fiber regions (Jeurissen et al. 2013). Fixel-based analysis was proposed to quantify the properties of white matter fibers in complex fiber geometry. The term “fixel” refers to a specific individual fiber population within a voxel (Raffelt et al. 2015, 2017) and is derived from white matter FODs as computed by the CSD method (Jeurissen et al. 2014). Recently, TractSeg was utilized to perform fixel-based white matter bundle segmentation in children with attention-deficit hyperactivity disorder (Fuelscher et al. 2021; Hyde et al. 2021) and older adults with metabolic syndrome (Andica et al. 2022).

## Technical limitations and future directions

Although the previous studies mentioned above have demonstrated the robustness of these approaches for evaluating brain disorders in patients ranging from children to older adults with brain disorders, the three tools have some technical limitations. AFQ only analyzes the diffusion measures at the central portion of the fiber tract. Therefore, marginal information related to the tract might not be assessed, and it is impossible to perform other quantitative measurements, such as fiber count and fiber bundle volume. Furthermore, TRACULA and TractSeg were trained using diffusion data from healthy young and middle-aged adults. A careful evaluation is needed to ensure the quality of the white matter segmentation in children or older patients, particularly in those with brain disorders.

Notably, it remains unclear how variations in data quality and acquisition affect white matter segmentation. The validation studies for these approaches were mainly performed using cross-sectional data with a relatively small sample size thus far. Furthermore, it is intriguing how race diversity will affect the segmentation results. Further studies using longitudinal, multi-site, multi-race data are required to demonstrate the robustness of these tools.

## Conclusion

This review describes the three most well-validated freely available diffusion MRI-based automatic white matter bundle segmentation tools, namely TRACULA, AFQ, and TractSeg. The pipelines of these approaches are robust with clinical quality diffusion MRI data and have many potentials. As aforementioned, previous studies have demonstrated the usefulness of these approaches for detecting white matter changes in brain development, brain disorders, and plasticity. TRACULA, AFQ, and TractSeg use different strategies to reconstruct white matter tracts. Therefore, the decision of which tool to adopt depends on the nature of the research aims, objectives, and questions. The three tools segment different parts of the white matter. TRACULA, AFQ, and TractSeg provide automatic segmentation of 42, 25, and 72 white matter tracts, respectively. Although the atlas includes a limited number of white matter tracts, the AFQ segmentation procedure applies a two-waypoint ROI method; thus, it can be modified to include additional fiber tracts (Yeatman et al. 2012). All methods described in this review enable tract profiling. It facilitates the analysis of white matter properties at specific positions within the tract profile. Furthermore, TractSeg makes it possible to train “your” model on “your” data, although it might be challenging (https://github.com/MIC-DKFZ/TractSeg#train-your-own-model). Finally, TRACULA provides a pipeline for longitudinal analysis of diffusion MRI measures in the white matter tracts.

## Data Availability

No datasets were generated or analyzed related to this review article.

## Abbreviations

AD: Axial diffusivity
AFQ: Automated fiber quantification
CSD: Constrained spherical deconvolution
CST: Corticospinal tract
DWI: Diffusion-weighted imaging
FA: Fractional anisotropy
FOD: Fiber orientation distribution
HCP: Human connectome project
MD: Mean diffusivity
MRI: Magnetic resonance imaging
RD: Radial diffusivity
ROI: Region of interest
TRACULA: TRActs constrained by underlying anatomy
TRACULInA: TRActs constrained by underlying infant anatomy
